# Dr. Morris W. Townsend

**Published:** 1902-04

**Authors:** 


Dr. Morris W. Townsend, of Bergen, N. Y., died at his home February 26, 1902, aged 75 years. He was a prominent physician in his region, served as a medical officer in the civil war, and attained considerable prominence as a surgeon. He was a member of local and state medical societies, and is survived by a son and a daughter, both of whom reside at Bergen.

